# Nucleotide sequence and results of test of adaptive evolution in the α-globin gene of octodontoid rodents

**DOI:** 10.1016/j.dib.2017.09.017

**Published:** 2017-09-18

**Authors:** I.H. Tomasco, N. Boullosa, F.G. Hoffmann, E.P. Lessa

**Affiliations:** a Departamento de Ecología y Evolución, Facultad de Ciencias, Universidad de la República, Iguá 4225, Montevideo 11400, Uruguay; b Department of Biochemistry and Molecular Biology, Mississippi State University, MS, USA; c Institute for Genomics, Biocomputing and Biotechnology, Mississippi State University, MS, USA

## Abstract

The data presented in this article are related to the research article entitled “Molecular adaptive convergence in the α-globin gene in subterranean octodontid rodents” (Tomasco et al., 2017) [1]. This article shows the nucleotide sequences of α-globin subunit gene of hemoglobin of several South American caviomorph rodents, including subterranean and fossorial species. These sequences are deposited in Genbank, with accession numbers ranging from MF169881 to MF169898. Of a total of 429 nucleotides analyzed (143 codons), 100 variable sites and 43 amino acid replacements were reported. In this article we also show the results of TreeSaap (Woolley et al., 2003) [2] and MEME (Murrell et al., 2012) [3], that identified some replacement changes as interesting for future studies of adaptive evolution in this large rodent radiation.

**Specifications Table**

**Table:** 

Subject area	*Biology*
More specific subject area	*Molecular Evolution*
Type of data	*Figures and tables*
How data was acquired	*Standard sequencing, and MEME (datamonkey.org), TreeSAAP software*
Data format	*Analyzed*
Experimental factors	*Edited sequences and use of Datamonkey server and TreeSaap Softwares*
Experimental features	*Standard sequencing from PCR products, edition and analyses.*
Data source location	*South America*
Data accessibility	*Genbank: accession from*MF169881*to*MF169898

**Value of the data**

• Adaptations to underground life have begun to be studied in some groups of mammals. In this article we show and analyze the nucleotide variation of endemic South American rodents, where two relatively recent occupation of subrerranean life allows an analyses within a robust phylogenetic framework, comparing fully subterranean species with their closest fossorial relatives.
• In these data, 43 amino acid replacements of the α-globin gene of 7 species of caviomorph rodents are reported for the first time, belonging to the family Ctenomyidae (Ctenomys rionegrensis, C. leucodon, C. sociabilis), Octodontidae (Spalacopus cyanus, Aconaemys fuscus, Tympanoctomys barrerae), Echimyidae (Proechimys longicaudatus) and Caviidae (Cavia aperea), which is a significant contribution to the diversity of these rodents, hitherto represented by three species (i.e.: Cavia porcellus, Chinchilla lanigera and Octodon degus).
• In a related a research article [1] we showed at least two sites under positive selection in the basal branch of Octodontidae. However, further studies are needed to identify the real role of those amino-acid replacement selected, such as biochemical and physiological assays activity in different conditions.
• We also show results of TreeSAAP and MEME software that could, that together with biochemical and physiological studies and/or ideally complemented with site-directed mutagenesis, to shed light on the effect of those changes. So, this results may be a starting point for such research, and generate new biological hypotheses for mutation studies and functional analyses.
• Finally, sampling the large number of tuco-tuco species would provide more statistical power and increase the ability to examine adaptive changes along multiple lineages.

## 1 Data

**Fig. 1:**
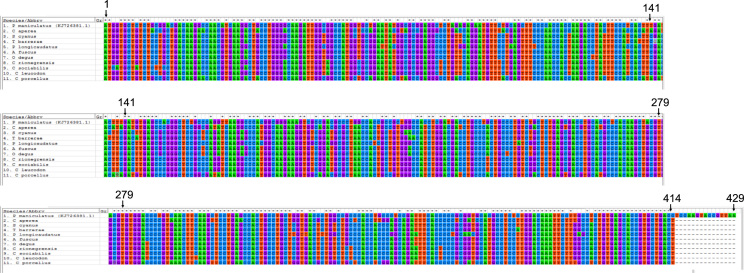
CDS of alpha globin gene of hemoglobin. These sequences were obtained in this study for several South American rodents. The sequence of *Peromyscus maniculatus* was added as reference. The position in the alignment was included.

**Fig. 2:**
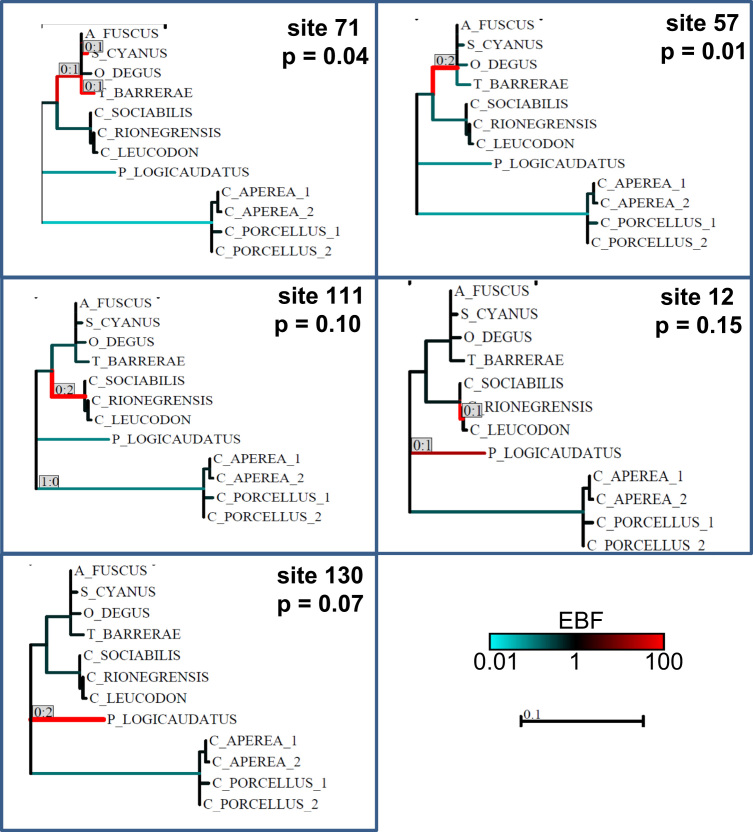
Summary of results obtained for each site found to be under episodic positive selection with MEME [3]. Codon site and probability are specified. Each branch is labeled with the pair Syn: NonSyn substitution counts. Branches with more total inferred substitutions are also drawn with thicker lines. Each branch is colored according to the magnitude of the Empirical Bayes Factor (EBF) for having *ω*>1 along that branch. Brighter reds higher EBF values (also see the scale bar).

A brief and conservative analysis of these results has been presented in a research article submitted to Gene journal [1]. In that study, the authors showed that at least sites 57 and 71 of the alpha globin gene show evidence of having been positively selected in the evolution of the octodontoid rodents. The door is left open for the analysis of other sites, for which the evidence was not so robust and are presented in this article. Particularly, summarized the complete cds sequences (Fig. 1) and all significant results obtained with MEME (Fig. 2) and TreeSAAP (Table 1) software. Particularly, MEME results show an interesting non-significant results is the site 111, that have nonsynonymous changes in the basal branch of subterranean Ctenomys (Fig. 2), and. TreeSAAP results show very radical changes in several branches of the tree (Table 1). The biological meaning of the changes in all these codon sites should be analyzed in the future in more details.

**Table 1:** Significant physicochemical amino acid changes among residues in alpha-globin gene identified by TreeSAAP.

Category 1 is the most conservative; categories from 6 to 8 are radical changes, being the 8 the most radical.

Codons highlighted in gray were suggested to be under positive selection by other methods.

## 2 Experimental design, materials and methods

We selected 10 caviomorph rodent species, including two independent subterranean lineages (Ctenomys and Spalacopus cyanus), three fossorial but non-subterranean relatives (Aconaemys fuscus, Octodon degus and Tympanoctomys barrerae), a non-fossorial spiny rat (Proechimys longicaudatus), and two species of Cavia (C. porcellus and C. aperea) used as outgroup. We chose a sample of 3 species from Ctenomys in an attempt to capture the diversity of lifestyles, molecular differentiation and habitat features [4].

Total DNA extractions were made from liver preserved in 95% ethyl-alcohol [5]. PCR amplification were carried out with two pair of primers specially designed for this study [1]. PCR products were purified and automatic sequencing from both ends was done by Macrogen. Inc. (http://www.macrogen.com), under BigDyeTM terminator cycling conditions in an ABI 3730xl Sequencer. The intron-exon structure and inspections of the lack of stop codons and in/dels that generate frameshift were checked. Aligned CDS are shown in Fig. 1.

Codon sites with evidence of have evolved under positive selection by different methods has been shown and summarized in [1]. Here we show additional results obtained with MEME [3] implemented in Datamonkey web server (http://www.datamonkey.org/, [6]) and TreeSAAP [2] software. MEME evaluates variation in dN/dS along branches, and then assess which sites contribute to variation in dN/dS, and the level for significance was 0.05 for the site and, conditional upon that, an Empirical Bayes Factor (EBF) greater than 20 for the branch or branches. TreeSAAP compares physicochemical properties of the amino acid changes observed. Nonsynonymous changes were considered to be the result of positive, destabilizing selection (from now on, “radical changes”) only if they met two stringent criteria: a) they were assigned to the most extreme categories of structural or functional changes (categories 6, 7 and 8 of [2]); and b) they were significant at the *p*<0.001. We ran ModelGenerator [7] to select a substitution model for each gene and reconstruct ancestral sequences in Baseml [8], and the model selected was REV [9]. The phylogeny considered for this study is shown in Fig. 2 and Table 1 and was obtained by pruning the comprehensive tree of octodontoid genera in [10], [11] and [4].
