# Corrigendum to “Nucleotide sequence and results of test of adaptive evolution in the α-globin gene of octodontoid rodents” [Data Brief 15 (2017) 517–521]

**DOI:** 10.1016/j.dib.2017.12.027

**Published:** 2017-12-16

**Authors:** I.H. Tomasco, N. Boullosa, F.G. Hoffmann, E.P. Lessa

**Affiliations:** a Departamento de Ecología y Evolución, Facultad de Ciencias, Universidad de la República, Iguá 4225, Montevideo 11400, Uruguay; b Department of Biochemistry and Molecular Biology, Mississippi State University, MS, USA; c Institute for Genomics, Biocomputing and Biotechnology, Mississippi State University, MS, USA

The authors regret that the article’s author list is incomplete and author Sophie Tandonnet is missing. The author list should have appeared as follows:

I.H. Tomasco,^a,*^ N. Boullosa,^a^ S. Tandonnet,^b^ F.G. Hoffmann,^c,d^ and E.P. Lessa^a^.

^a^ Departamento de Ecología y Evolución, Facultad de Ciencias, Universidad de la República, Iguá 4225, Montevideo 11400, Uruguay.

^b^ School of Life Sciences, University of Warwick, Coventry CV4 7AL, UK.

^c^ Department of Biochemistry and Molecular Biology, Mississippi State University, MS, USA.

^d^ Institute for Genomics, Biocomputing and Biotechnology, Mississippi State University, MS, USA.

The authors would like to apologise for any inconvenience caused.

